# The road to achieving epidemic-ready primary health care

**DOI:** 10.1016/S2468-2667(23)00060-9

**Published:** 2023-04-27

**Authors:** Thomas R Frieden, Christopher T Lee, Mohammed Lamorde, Marci Nielsen, Amanda McClelland, Viroj Tangcharoensathien

**Affiliations:** a Resolve to Save Lives, New York, NY, USA; b Infectious Diseases Institute, Makerere University, Kampala, Uganda; c International Health Policy Program, Amphur Muang, Nonthaburi, Thailand

## Abstract

Millions of avoidable deaths arising from the COVID-19 pandemic emphasise the need for epidemic-ready primary health care aligned with public health to identify and stop outbreaks, maintain essential services during disruptions, strengthen population resilience, and ensure health worker and patient safety. The improvement in health security from epidemic-ready primary health care is a strong argument for increased political support and can expand primary health-care capacities to improve detection, vaccination, treatment, and coordination with public health—needs that became more apparent during the pandemic. Progress towards epidemic-ready primary health care is likely to be stepwise and incremental, advancing when opportunity arises based on explicit agreement on a core set of services, improved use of external and national funds, and payment based in large part on empanelment and capitation to improve outcomes and accountability, supplemented with funding for core staffing and infrastructure and well designed incentives for health improvement. Health-care worker and broader civil society advocacy, political consensus, and bolstering government legitimacy could promote strong primary health care. Epidemic-ready primary health-care infrastructure that is able to help prevent and withstand the next pandemic will require substantial financial and structural reforms and sustained political and financial commitment. Governments, advocates, and bilateral and multilateral agencies should seize this window of opportunity before it closes.

## Introduction

The COVID-19 pandemic has revealed—and increases the potential to address—life-threatening weaknesses in primary health-care systems in most countries. During the pandemic, detection of spread of COVID-19 and other infectious diseases was delayed, many primary health-care systems were unable to vaccinate patients within care facilities, provision of medications that could reduce hospital admission was insufficient, health-care workers were inadequately protected, and ongoing primary care was disrupted.^1^ Framing and enhancing primary health care to be ready for an epidemic can be both a strong political argument for increased support for these facilities, and can expand primary health care capacities to address needs that became more apparent during the pandemic. We propose attributes of and means to make progress towards preparing primary health-care facilities for an epidemic as part of an effective societal response to health threats, but which does not substitute for robust public health functions and a multisectoral response involving governmental and non-governmental entities.

Primary health care can be broadly conceptualised to include advocacy for social change, addressing the social determinants of health, and increasing health equity. In this Viewpoint, we focus on clinical systems and their interaction with public health. Primary health care delivered by trusted health-care providers follows evidence-based guidelines and is continuous, coordinated, team-based, and patient-centred.^2^

Although countries have long stated their commitment to improving primary health care, three quarters of people in the world—including at least 85% of people in low-income and middle-income countries—still do not benefit from accessible, affordable, and effective primary health care.^3^ Global investments in immunisation, maternal and child health, HIV, tuberculosis control, and malaria programmes introduced substantial new funding into health-care services, but in most countries have not yet led to strong, sustainable, and resilient primary health-care systems.^4^, ^5^

## Components of an epidemic-ready primary health-care system

Although traditional definitions of primary health care include components that would help detect and respond to epidemics, this has not generally been a focus of primary health-care advocacy or development, and aspects of epidemic-ready primary health-care elements (eg, infection control, disease reporting, continuity of operations in an emergency) have sometimes been neglected by both governments and funding organisations. Epidemic-ready primary health care is the intersection of clinical services and public health along with the prevention, detection, and response to epidemics. Many outbreaks are detected because a patient has sufficient trust to promptly seek care from a clinician and an alert clinician with strong links to the public health system suspects and reports the case. An epidemic-ready primary health-care system can protect and improve health as follows, and is summarised in the panel.

Panel **Components of epidemic-ready primary health care**

**Diagnosis and reporting**

• Prompt detection and reporting of notifiable diseases and unusual health events as a result of a high index of suspicion
• Access to point-of-care diagnostics and a quality-assured and timely laboratory network

**Treatment and care**

• Maternal, reproductive, infant, and child health care
• Treatment of common symptomatic conditions (eg, cough, rash, diarrhoea, pain) so that patients and their families trust and can rely on their provider
• Treatment of common infectious diseases (eg, sexually transmitted infections, tuberculosis, HIV, malaria, pneumonia)
• Provision of mental health and psychosocial services
• Detection, effective treatment, and monitoring of hypertension, diabetes, and cancer, thereby increasing the health resilience of the population

**Prevention and response**

• Vaccination that reaches all or nearly all patients for which each facility is responsible
• Surveillance systems which support timely detection, care, vaccination, and containment of outbreaks, appropriately targeted to populations at highest risk

**Staffing**

• Adequate training including public health competencies, compensation, protection, and support of all facility staff
• Community health workers contribute to task-sharing, team-based care, and community outreach
• Designated focal points at each facility for infection prevention and control, and for disease reporting and coordination with public health agencies

**Infrastructure**

• Stable and reliable logistics and supply chains to ensure ready availability of medications, diagnostics, and personal protective equipment
• Sufficient laboratory system capacity to promptly identify pathogens and facilitate rapid diagnosis and feedback to clinicians
• Sufficient and clean water and sanitary waste disposal services
• An effective information system for patient care and programme management which tracks the community-wide impact of clinical services among all people for whom each facility is responsible

**Infection prevention and control**

• A designated individual who serves as a focal point for infection prevention and control and follows national guidelines for administrative, operational, and personal protection policies
• Systematic monitoring and improvement of infection prevention and control, including tracking infections associated with health care among staff and patients

**Continuity of operations**

• Specific plan to continue essential services during societal disruption (eg, floods, pandemic, insecurity)

**Linkages**

• Effective linkages via referral, including bidirectional information flow, between primary health care and hospital and specialty care
• Community engagement, through both formal and informal means, to increase awareness of and community feedback to each facility
• Connection with available social services to address patient needs

**Close coordination with public health agencies**

• Tracking of health indicators for the entire empanelled or geographically assigned population
• Strong linkage between primary health care and public health to facilitate rapid detection, notification, treatment, vaccination, communication, and community engagement
• Partnership to support community-wide interventions to promote healthy environments so that the default choices are the healthy choices (eg, safe water and sanitation, tobacco and alcohol use prevention policies, clean air including through cleaner fuels for cooking and heating, safe streets to reduce road traffic injuries and promotion of walking and cycling, and healthy nutrition)

The first benefit of an epidemic-ready primary health-care system is prompt detection of outbreaks through a high index of clinical suspicion, access to accurate and timely laboratory tests, and close communication with local public health authorities. Timely and complete reporting to public health entities enables real-time tracking of community-wide disease trends, which in turn enables public health authorities to alert clinicians of trends and improve case detection.

An epidemic-ready primary health system will help with treatment and care for common symptomatic conditions as well as infectious and non-communicable diseases. Treating causes of ill health increases patient trust in and use of the system and thereby increases the likelihood that an outbreak or newly emerging disease will be promptly recognised. Treatment of HIV, tuberculosis, malaria, non-communicable diseases, and other conditions that are usually detected in primary health care facilities increases the sustainability and accessibility of these high-impact services. Prepared primary health-care systems will increase prevention and response to infectious disease outbreaks through vaccination,^6^ patient education, and treatment, all coordinated with local public health messaging, programmes, and policies. In areas such as regions of Africa and Asia where zoonotic pathogens are particularly likely to cause human outbreaks, close collaboration with One Health programmes is important to improve prevention, early detection, and control of animal to human transmission.^7^

Additionally, mental health services would support people who face displacement, disease, or death and are coping with the aftermath of an epidemic or other emergency. Infrastructure would become more robust and sufficient, including logistics with stable supply chains, laboratory systems, and effective information systems. An effective primary health-care system would include a user-friendly, accurate, and useful information system. Staff, including community health workers, would receive sufficient training and subsequently easily integrate into primary health-care systems. A safe environment would be created for health-care workers and patients through infection prevention and control, including systematic monitoring and improvement, and there would be a designated focal point to increase adherence to national policies and guidelines.

Furthermore, financial protection for families and the efficiency of the health-care system would increase. Improved health and less illness and disability can increase productivity, reduce patient and governmental spending on expensive hospital admissions and specialty care, and mitigate economically devastating epidemics.^8^ There would be practical plans to continue essential services during disruptions including epidemics, civil unrest, and other emergencies.^9^ Effective linkages among primary health care, hospitals, specialty care, public health, and communities that facilitate referrals to and from hospital and specialty care; integration with public health; and trust-building with communities would all improve. Patients referred from primary health care settings would be expeditiously attended to by specialists or in hospitals, and patients diagnosed in hospitals would benefit from easier transfer to facilities and effective management at the primary health-care level.

Improving care throughout the life course would also improve population resilience. This includes children who are vaccinated, protected against enteric infections, supported to have healthy nutrition, and treated promptly when ill; adults whose common conditions such as hypertension and diabetes are prevented wherever possible and treated effectively; older adults who are supported to remain independent; and patients with terminal illness who receive appropriate palliative care. If primary health-care settings become epidemic-ready, all of the aforementioned population groups would be less likely to acquire, spread, and die from infections resulting from outbreaks.

One of the most important lessons of the COVID-19 pandemic is that trust is fundamentally important to increase adherence to public health and social measures.^10^ Provision of effective primary health care builds trust between individuals and providers and between communities and the health-care system, especially when care is provided in a culturally sensitive way by well-trained and adequately paid staff. Care that is readily accessible and reliable with minimal or no out-of-pocket patient costs for essential medications and necessary laboratory tests is particularly important for marginalised and underserved communities, which are at higher risk of spread of infectious disease because of higher disease rates and less access to health care and lower levels of trust, leading to delayed recognition of disease outbreaks.

## How to advance towards an epidemic-ready primary health-care system

The extraordinary costs of the COVID-19 pandemic resulted in unprecedented attention from finance and health ministers and political leaders, providing a unique but brief opportunity to advocate for and obtain increased and sustained investments in public health and primary health care.^11^ In response to the COVID-19 pandemic, WHO has recommended strengthening primary health care to achieve universal health coverage and global health security through: increasing political commitment and leadership, promoting innovation in public and private sectors, ensuring adequate and competent health-care staffing, expanding much needed financing, and improving infrastructure and logistics for safe delivery of primary health-care services.^12^

However, the pandemic alone will not be enough to catalyse these improvements: memory of it is fading, lessons that should be learned might be forgotten, and many government budgets will be even more constrained and face competing priorities. Strengthening epidemic-ready primary health care will require a fundamental shift in advocacy for health-care governance, financing, and integration with public health. Progress will only be possible through action based on understanding of who benefits and loses from stronger primary health care, effective coalition-building that begins with existing organisations and institutions, and strategic advocacy to influence policy makers.^13^, ^14^

Understanding the primary reasons for the low priority of primary health care in most countries’ health-care financing policies is important. The root causes are both political and economic. Patients, staff, facility operators, owners, and investors who benefit from specialised, acute, and hospital-based care have more political power, and capital-intensive spending on hospitals, medical technologies, and expensive medical equipment creates pressure to increase spending on specialised care. Furthermore, health professional training continues to encourage specialisation.^15^ As a result, primary health-care providers earn far less than specialists in most countries; allied health workers including nurses, nurse practitioners, community health workers, and pharmacists are underpaid and in short supply; and few systems provide meaningful financial incentives for primary health-care systems to preserve and improve health and protect from pandemics.

Because few low-income and middle-income countries have established robust primary health-care systems, definitive evidence on how to make progress is limited. Three complementary approaches are promising as strategies to increase support and funding for epidemic-ready primary health-care systems. The first is for countries to be explicit about core services which can be funded at existing levels of spending and to provide transparent information on performance of service delivery (eg, vaccinations, community viral load suppression, and control of blood pressure to <140/90 among all people living in the area with hypertension). By contrast, in many countries, de facto rationing occurs: in theory, all medications and services are available at no or low cost but, in practice, shortages of workers, medications, and functioning equipment constrain care.^16^

A more effective and equitable approach, although politically more challenging, is for a transparent, government-led, multi-stakeholder consensus process to determine a core set of primary health-care services that will be consistently available throughout the country and transparent reporting on the level at which services are provided, with agreement that additional services will be offered as fiscal capacity allows, as has been done for example in Chile, Malawi, and Ethiopia.^17^, ^18^, ^19^ Technical rigour in selection of core services and care pathways, along with accurate real-time transparent monitoring, decentralisation of care to the primary health-care level, and better management and administration of programmes can substantially improve health outcomes.^20^ Governmental revenue generation that is progressively structured and pooled for cross-subsidisation can improve equity in service delivery. In Ghana, analysis indicates that a primary health-care approach might be most feasible in the short term to bridge the gap between the official service package and the cost of implementing this package.^21^ Because the private health-care sector is an important source of treatment in most low-income and middle-income countries, improved quality, oversight, and integration of health care provided by this sector will be important and can extend or complement public sector services.

A second potential means of strengthening primary health care is for countries to increase the amount and efficient use of all financial sources, including expeditious efforts to realign external funding from global organisations that fund disease-specific programmes. Governments can insist that disease-specific funders and bilateral and multilateral agencies coordinate investments, identify complementarity, and invest in primary health care to enable specific programme targets. These external resources can be increasingly channelled to strengthen components of epidemic-ready primary health care (eg, facility improvements, infection prevention and control, primary health-care information systems, and multi-pathogen specimen transport and laboratory networks). Because countries that implement epidemic-ready primary health care improve the continuity of essential service delivery during epidemics and other disruptions, they are more likely to achieve specific programme targets (eg, for immunisation, HIV, tuberculosis, and malaria); countries can use this fact to insist that disease-specific programmes better support epidemic-ready primary health-care systems and reduce donor-induced system fragmentation.

Domestic funding for recurrent primary health-care expenditures, including workforce and essential medicines and diagnostics, must be predictable and sustained to gain community and health-care worker trust. Efficiencies can be accomplished in many places through improved management of procurement, payroll, and contracting; improved hospital management; and establishing optimal pathways for diagnosis and treatment. Some countries such as the Dominican Republic have successfully reinvested cost savings from improved efficiency into the health sector.^22^, ^23^ Increased funding can be facilitated, for cases in which this can be done without risk of reprisals, by advocacy from health-care worker organisations, patient organisations, and other civil society organisations. Budgets linked to achievement of specific outcomes established in the core service package can increase the provision of these services. Improved health sector performance has health, social, and economic benefits, which can help justify increased funding. Optimising the use of external and national resources might require policy coordination, including mapping and forecasting external and domestic funding sources.

A third area of focus to improve primary health care, albeit based on limited experience in practice in low-income and middle-income countries, could be to change the structure of domestic financing of health-care services, with increased payment for and strategic purchasing of high-quality, accessible primary health-care systems. Changing payment policies requires strategic political action and broad engagement by ministries of health and finance, community and civil society groups, health-care providers and organisations, donors, and technical agencies. Analysis of the political economy should underpin strategies from the outset, with the goal of ensuring broad and sustained stakeholder engagement and support for primary health care. Although a wide range of initiatives can improve quality, aligning the overall financial structure of care with improved patient outcomes is likely to be the most effective and sustained means of improving care.^14^ In Brazil, fixed per capita funding supplemented by additional funding based on government priorities enlarged the scope of primary health-care services delivered by improving access to health care, reducing social inequalities, and improving health outcomes.^24^

Fee-for-service payment is a key factor in supplier-induced demand, cost escalation, and inefficiency.^25^ Paying health-care teams adequately for empanelled patients via capitation (per patient per month or quarter for a defined set of primary health-care services, rather than for each visit or procedure performed) can incentivise coordinated, team-based care accountable to a specific population and should be the cornerstone of primary health-care financing wherever feasible.^14^ Capitation as the foundation for primary health-care payments in Mongolia, Thailand, and Estonia has led to improved equity and cost containment without compromising service quality.^26^, ^27^

In addition to capitation, two additional structural financing components that appear necessary are core and stable funding for epidemic-ready primary health-care infrastructure and carefully designed incentives to improve care of high-priority conditions. Infrastructure support includes funding and technical assistance for surveillance and capacity for rapid response to outbreaks, the health-care workforce (particularly in rural and low-income urban communities and other neglected areas), training, support for health-care worker safety, and data systems. Incentives to improve outcomes, as have been implemented in Rwanda,^28^ should lead to demonstrable and substantial health improvements such as improved vaccination rates, higher coverage of reproductive health needs, and control of hypertension.

These approaches can result in a virtuous cycle of strengthening for primary health care, as has been observed in, for example, Thailand and Costa Rica. When epidemic-ready primary health-care services are delivered by trusted local providers, these services are more likely to be trusted by communities and, as a result, become more politically popular. This increased popularity can lead to increased support from policy makers, who further increase funding. As government funding grows, health services continue to improve. As this virtuous cycle repeats, momentum and public support for primary health-care services increases, overcoming inertia and eventually achieving escape velocity—transcending partisanship and gaining recognition and support from a wide spectrum of political parties as a national priority and prized resource.^29^

Four countries with strong primary health-care systems illustrate political routes to improved governance, increased funding, and healthier outcomes. In Thailand, the risk of insurgency, along with activist doctors and other health-care workers, became potent political forces leading the government to increase funding for primary health care.^30^ Recruitment for health professional education through a special track for local students resulted in higher levels of competency and staff who served local communities for longer.^31^ In Costa Rica, a country without strong entrenched interests, social democratic leadership emerging from a civil war created a coalition among all major political parties to promote basic health care and education to prevent revolutionary upheavals. This coalition led to recognition of health and other social goods as national rather than partisan objectives and resulted in improvements in care delivery that have continued for several decades.^32^ In Cuba, political legitimacy of the government rests substantially on its commitment to providing effective health and education services;^33^ improved health in Sri Lanka also largely depends on widely accepted and universally available free education and health services.^34^

These disparate potential routes to stronger political support for primary health care—health-care worker advocacy, political consensus, and bolstering the legitimacy of the government—can be appropriately and differentially applied in specific country contexts, building on existing institutions, organisations, advocacy, and political leadership to advance primary health care. Individual and organised advocates, including health-care workers, community-based organisations, patients and their families, politicians, and others can be decisively important for progress.^35^

## Barriers to improving primary health care and potential solutions

After half a century of calls to strengthen primary health care ranging from Alma Ata to Astana and more, as well as recent calls to improve primary health care in the wake of the COVID-19 pandemic,^1^ it is important to recognise barriers that must be overcome. More than 20 years after the African Union Abuja declaration called on all countries in the region to invest at least 15% of government spending on health, few countries have achieved that target, despite the positive economic impact of such spending.^36^ Furthermore, government budgets are so constrained in many countries that dedicating even 15% of spending on health might be insufficient to provide basic health services, particularly because most government funds spent on health go to secondary and tertiary care.^37^

The evidence base is weak concerning which specific interventions countries should implement at the primary health-care level to improve resilience, as is documentation that this type of care can increase the efficiency of governmental public health systems.^38^ Implementation would optimally be done with real-time evaluation and optimisation, overseen by a multi-stakeholder group including broad participation from inside and outside of government, to rigorously analyse and steadily increase the health benefit of primary health-care services.

Private sector care—including over-the-counter sales of antibiotics and other medications by pharmacies—and care by traditional, non-licensed practitioners, is more accessible than government-operated primary health care in many low-income and middle-income countries, but often provides poor-quality care and risks missed outbreaks and development of antimicrobial resistance. Improved access to and quality of publicly funded primary health care can reduce patient reliance on unregulated services, and public sector regulation of and collaboration with privately provided health care, including private laboratories and non-licensed practitioners, can improve quality and facilitate reporting of notifiable diseases.^39^

Shortages of personal protective equipment during the COVID-19 pandemic highlighted gaps in manufacturing capacity and supply chains which hamper the epidemic readiness of primary health care. Health-care worker advocacy, establishment of designated focal points at the facility level, and focused infection prevention and control units at the national level, along with improved product design, local manufacturing, supply chain management, and global financing, can improve availability.

Laboratory diagnostics can be a bottleneck for outbreak detection, notification, and response, and require investments in supplies, specimen transport and referral systems, quality management, biosafety, and effective information systems. About a quarter of health facilities worldwide do not have adequate access to clean water or sanitation.^40^

Primary health-care information systems can often show actual or perceived lack of utility, insufficiently trained operators, and poor internet connectivity.^41^ Maintaining effective facilities and services is made more difficult by shortages of hard currency, difficulty enforcing warranties, procurement and supply inefficiencies and, in some areas, corruption in the procurement and maintenance of medical equipment. These issues need to be addressed through information systems that make on-the-ground realities apparent to decision makers and, ideally, the public; management improvements that optimise use of existing funds; and strategic resource mobilisation to fund essential services.

Because hospital outpatient departments in many countries are more likely than are primary health-care systems to have trained staff, equipment, and laboratories able to accurately diagnose and treat acute conditions, patients seeking primary care services in many low-income and middle-income countries often bypass the primary health-care system altogether. For credibility and efficiency of primary health-care systems, effective two-way referral is essential—patients must feel that they benefit from local primary health-care services not only at that facility but also for specialty and hospital services when these are required.

There is an estimated global shortage of more than 15 million health workers, mostly in low-income settings and primarily because health workers are undercompensated.^42^ Higher salaries and improved working conditions will be important to attract and retain primary health-care staff. Community health workers can help alleviate staffing shortfalls through task-sharing, team-based care, and community outreach and, with additional training and pay, can graduate to provide increasingly complex care.

Improving the skills of the primary health-care workforce is a formidable challenge that might only be addressed in part by resolving financial challenges. Many public and private sector health workers lack training and education for disasters and feel unprepared when events occur.^38^ Countries should train primary health-care personnel, including front-line clinicians and community health workers, to recognise patients or events suggestive of disease outbreaks and should track and report on the implementation of such training. Due to resource limitations, some countries prioritise training of surveillance personnel, health workers at secondary and tertiary care levels, or only public sector health workers. However, doing so creates a gap at the point of detection that must be addressed with additional resources and locally effective training models and technology. Pre-service curricula for health workforce training must be revised to meet epidemic prevention, detection, and response needs at the primary health-care level.

## Conclusion

Primary health-care systems in most countries are far from epidemic-ready. Progress is likely to be stepwise and incremental, advancing where and when opportunity arises. Primary health care needs to be strengthened to improve health system and community resilience, but this can only be accomplished with a fundamental shift in our approach to health-care governance, financing, and integration with public health. Health, finance, and political leaders can realign existing resources and increase funding, promote accountability, and advocate to close gaps in achieving epidemic-ready primary health care. Civil society groups can advocate for and track progress, and bilateral and multilateral partners can increase their funding of system-wide improvements which also support programme-specific goals. Country-specific political analysis and sustained advocacy can result in increased and more efficiently used funds and can change the incentives created by health-care financing to optimise health outcomes and health protection.

Improved primary health care is a cornerstone for progress toward universal health coverage, achievement of the Sustainable Development Goals, and global health security.^43^, ^44^, ^45^, ^46^, ^47^ A strong and resilient epidemic-ready primary health-care infrastructure could be a landmark achievement of our pandemic recovery, but will require rapid action while the disruption of the pandemic can still motivate change to achieve the financing and structural reforms needed for these systems to improve both provision of routine care and preparation for health emergencies.

### Search strategy and selection criteria

A literature search was done to inform development of elements of this Viewpoint. Publications in English were identified from PubMed and from Google internet searches using the following key search terms: “COVID-19”, “health care financing”, “health care governance”, “health care service continuity”, “health care worker compensation”, “health care worker safety”, “HIV and primary health care”, “immunization and primary health care”, “infectious disease outbreak prevention”, “pandemic preparedness”, “population resilience”, “primary health care”, “primary health care system strengthening”, “public health”, and “public health infrastructure strengthening”. These seaches were conducted on Oct 30, 2022, and again on Jan 28, 2023. No date restrictions were applied to the search, although we used the most recent relevant citations where applicable. Publications identified were screened for relevance based on their titles and abstracts. Additional relevant articles were identified from references of selected publications, and relevant publications of major organisations including WHO and the World Bank were also reviewed. No restrictions were placed on date of publication, although most relevant publications identified were published immediately before or during the COVID-19 pandemic.

## Declaration of interests

We declare no competing interests.
